# Genome-wide DNA methylation analysis revealed stable DNA methylation status during decidualization in human endometrial stromal cells

**DOI:** 10.1186/s12864-019-5695-0

**Published:** 2019-04-29

**Authors:** Ryo Maekawa, Isao Tamura, Masahiro Shinagawa, Yumiko Mihara, Shun Sato, Maki Okada, Toshiaki Taketani, Hiroshi Tamura, Norihiro Sugino

**Affiliations:** Department of Obstetrics and Gynecology, Yamaguchi University Graduate School of Medicine, Minami Kogushi 1-1-1, Ube, 755-8505 Japan

**Keywords:** DNA methylation, Decidualization, Endometrial stromal cells, RNA expression, And histone modification

## Abstract

**Background:**

During decidualization in endometrial stromal cells (ESCs), expressions of a number of genes and epigenetic modifications of histones are altered. However, there is little information about whether DNA methylation, which is another epigenetic mechanism, also changes during decidualization. Here, we examined the genome-wide DNA methylation profiles in ESCs during decidualization and their associations with the changes of gene expressions and histone modifications.

**Results:**

ESCs were incubated with estradiol and medroxyprogesterone acetate for 14 days to induce decidualization. The genome-wide DNA methylation profiles were compared between the non-decidualized ESCs and the decidualized ESCs. Of 482,005 CpGs, only 23 CpGs (0.0048%) showed different DNA methylation statuses. The DNA methylation statuses of the differentially expressed genes and the regions with different histone modifications (H3K4 tri-methylation and H3K27 acetylation) were also compared between the ESCs. In the upregulated and downregulated genes in decidualized ESCs, DNA methylation statuses around the promoter region of the genes did not significantly differ between the ESCs. In the regions with different histone modification, DNA methylation statuses did not differ between the ESCs. The differentially expressed genes and the differential histone modification regions were hypomethylated.

**Conclusions:**

Culturing ESCs with estrogen/progesterone did not distort the physiological pattern of DNA methylation, although mRNA expression and histone modifications were dynamically altered. A genome-wide DNA methylation analysis revealed stable DNA methylation statuses during decidualization in human endometrial stromal cells. DNA hypomethylation is maintained for the variable changes of histone modifications and gene expression during decidualization.

**Electronic supplementary material:**

The online version of this article (10.1186/s12864-019-5695-0) contains supplementary material, which is available to authorized users.

## Background

Human endometrial stromal cells (ESCs) show cyclic changes during the menstrual cycle, which is regulated by estrogen and progesterone. Especially, the process in which fibroblastoid stromal cells of the estrogen-primed endometrium shift into decidual cells by progesterone is called decidualization. Decidualization is crucial for embryo implantation and maintenance of pregnancy [1, 2].

The expressions of a number of genes change during decidualization, suggesting that decidualization is accompanied by dramatic changes of cell functions. Gene expression includes a change of chromatin structure, which can be regulated by epigenetic mechanisms such as DNA methylation and histone modifications [3, 4]. Recently, we found that active marks of histone modifications such as histone H3K4 tri-methylation (H3K4me3) and histone H3K27 acetylation (H3K27ac) dramatically changed in ESCs during decidualization, and these histone modifications were associated with the changes of gene expression [5].

DNA methylation is a well-characterized epigenetic mark. DNA methylation, which occurs at CpG sites, interrupts the recognition and binding of transcription factors [6–10], recruits methyl CpG-binding proteins that interact with transcription repressors [7, 11], and induces chromatin condensation via histone modification changes [7, 12]. DNA hypermethylation at promoter regions is associated with gene silencing, while DNA hypomethylation is associated with gene activation [7]. In most of the genes including housekeeping genes, the promoter regions are DNA hypomethylated in all types of cells/tissues. On the other hand, there are genes whose DNA methylation statuses are specific to each cell/tissue and have important roles in determining the cell/tissue-specific gene expression profiles [7, 13, 14]. To better understand decidualization, it is of interest to know 1) whether the DNA methylation profiles change and contribute to the changes of gene expression profiles in ESCs during decidualization and 2) how two major epigenetic marks, DNA methylation and histone modifications, are intertwined with each other during decidualization.

In the present study, we compared the genome-wide DNA methylation profiles between non-decidualized ESCs and decidualized ESCs. We next compared the DNA methylation statuses of the genes whose mRNA expression profiles differed between the non-decidualized ESCs and the decidualized ESCs. Since histone modifications are known to interact with DNA methylation [15–18], we compared the DNA methylation statuses of the regions in which histone modification (H3K4me3 and H3K27ac) statuses were different between the non-decidualized ESCs and the decidualized ESCs.

## Results

### Genome-wide DNA methylation profiles of ESCs

**Fig. 1:**
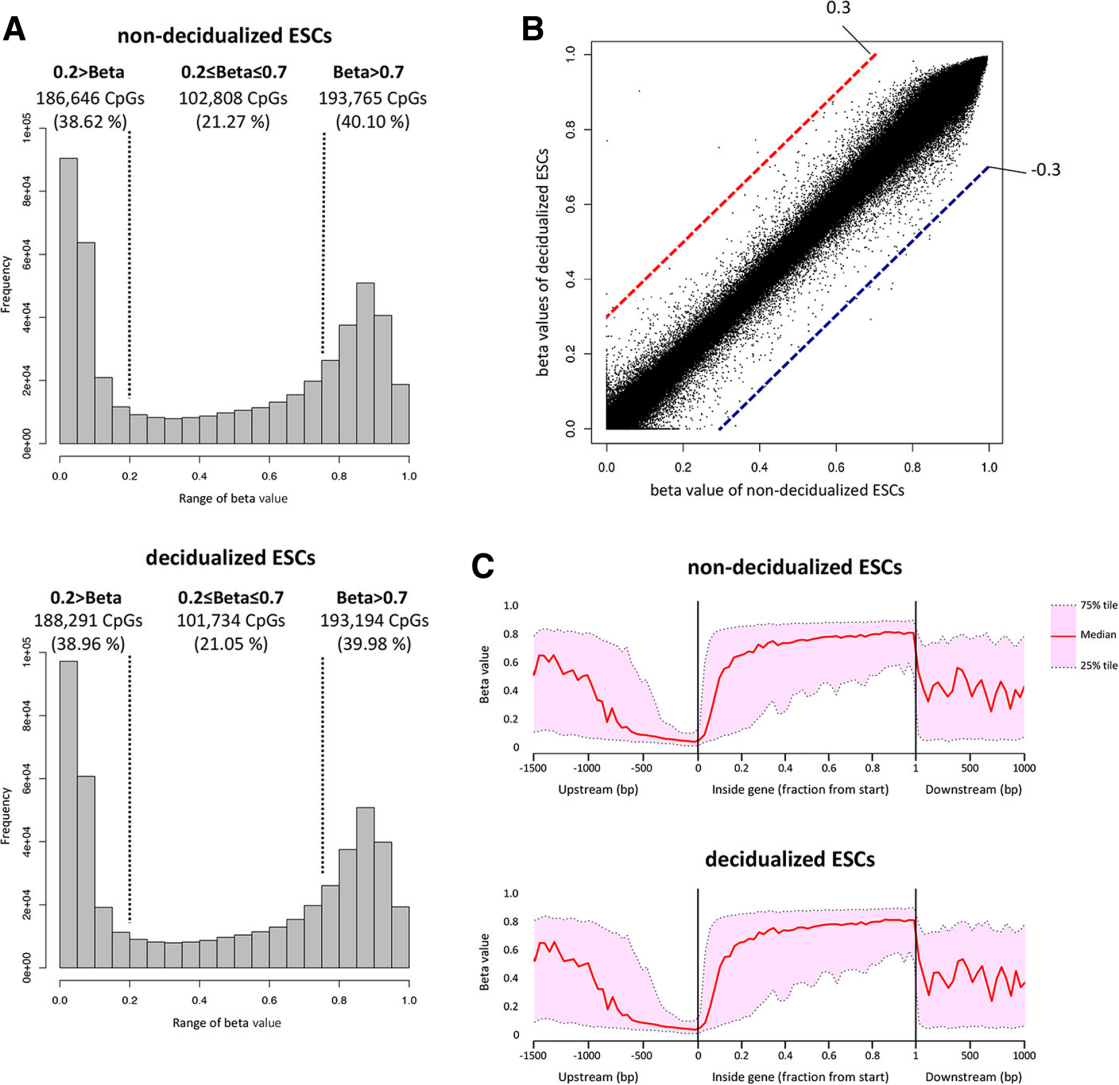
Genome-wide DNA methylation profiles of the non-decidualized and decidualized ESCs. **a**: Histograms of the DNA methylation levels (beta-values) of 482,005 CpGs in the non-decidualized and decidualized ESCs. In each range of beta-values (0.2>, 0.2 to 0.7, and 0.7<), the frequencies and the ratios of the methylated CpGs to all CpGs are shown. **b**: Scatterplot of the beta-values in the non-decidualized and decidualized ESCs. The CpGs are plotted according to the beta-values of the non-decidualized (horizontal axis) and decidualized ESCs (vertical axis). Dashed lines show minimum beta-value changes of 0.30. Red dashed lines indicate the cut-off beta-values for the CpCs which have increased beta-values during decidualization while blue dashed lines indicate the cut-off values for the CpGs which have decreased beta-values. **c**: DNA methylation level vs. gene position in the non-decidualized (top) and decidualized ESCs (bottom). The DNA methylation profiles of upstream region (from − 1500 bp to transcription start site), gene body region (from transcription start site to transcription end site) and downstream region (from transcription end site to + 1000 bp) of the genes are shown. In the gene body region, the distance from transcription start site to transcription end site was rescaled into the range of [0–1] as previously reported [18]. Median (50%), 25 and 75% tile beta-values are plotted in the non-decidualized and decidualized ESCs (**c**). Vertical and horizontal lines show beta-values the position relative to the transcription start site

To assess the overall patterns of DNA methylation, we examined the DNA methylation levels of 482,005 CpGs in the ESCs. The average DNA methylation levels (beta-values) of the CpGs in the non-decidualized and decidualized ESCs were 0.468 ± 0.364 and 0.465 ± 0.366, respectively. As shown in Fig. 1a, in the non-decidualized and decidualized ESCs, the majority of CpGs were hypomethylated (beta-value< 0.2; 38.62 and 38.96%, respectively) or hypermethylated (beta-value > 0.7; 40.10 and 39.98%, respectively), and the remaining CpGs had intermediate levels of DNA methylation (0.2 ≤ beta-value ≤0.7; 21.27 and 21.05%, respectively). There was no significant difference in the distribution patterns of the methylated CpGs between the decidualized and non-decidualized ESCs.

To assess the number of the CpGs that were differentially methylated between the non-decidualized and decidualized ESCs, the beta-values of all CpGs in both ESC types were plotted in a scatter plot (Fig. 1b). The scatter plot showed a linear distribution pattern, indicating that there were few differences in the DNA methylation levels between the two groups. In fact, when the cut-off of the beta-value is more than 0.3, which is generally accepted, only 23 CpGs were differentially methylated (Table 1).

**Table 1:** The number of the differentially methylated CpGs

Cut-off of beta value	0.15 <	0.30 <
Total	582 (0.1203%)	23 (0.0048%)
Hypomethylated CpG sites	210	8
Hypermethylated CpG sites	372	15

Human endometrial stromal cells were treated with or without E and MPA for 14 days. DNA methylation statuses of the non-decidualized and decidualized ESCs were analyzed by Illumina Infinium HumanMethylation450 BeadChip. The number of the differentially methylated CpG sites was calculated when the difference of DNA methylation levels (beta-value) between the two types of ESCs was more than 0.15 and 0.3

We compared the DNA methylation profiles based on the gene structures between the ESCs. In the present study, we obtained the DNA methylation statuses of 482,005 CpGs among 41,204 genes. The CpGs were grouped into three regions: upstream region (from − 1500 bp to transcription start site), gene body region (from transcription start site to transcription end site) and downstream region (from transcription end site to + 1000 bp) of the genes. These regions included 253,082, 564,325 and 22,012 CpGs, respectively. As shown in Fig. 1c, the DNA methylation patterns of the three regions were very similar between the two ESC types, and the DNA methylation status was hypomethylated in the promoter region (− 500 bp to transcription start sites) in the both ESCs.

### DNA methylation profiles of differentially expressed genes

**Fig. 2:**
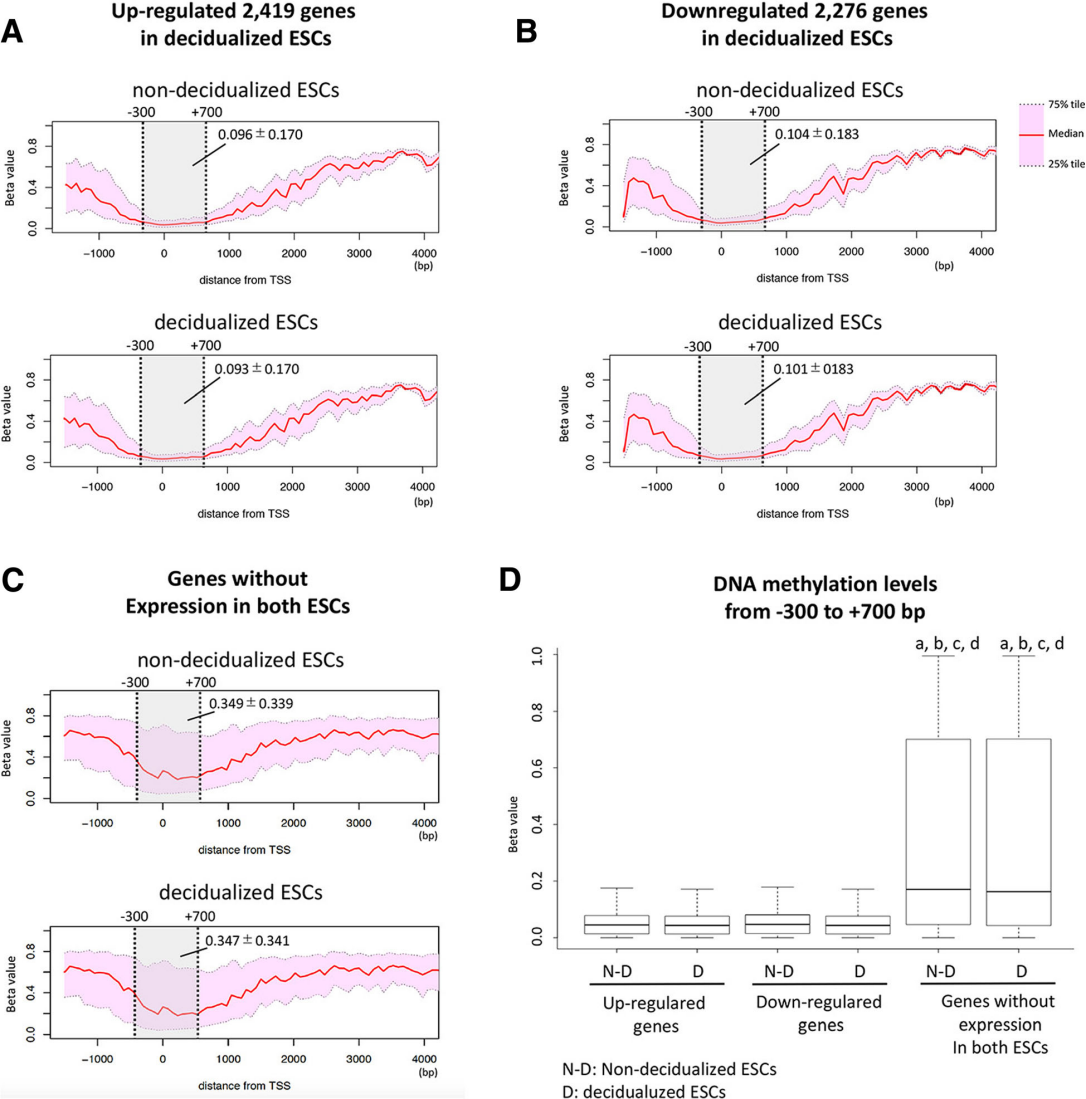
DNA methylation status of differentially expressed genes between the non-decidualized and decidualized ESCs. **a** and **b**: Median (50%), 25 and 75% tiles of beta-values of up-regulated 2419 genes (**a**) and down-regulated 2276 genes (**b**) are shown in the non-decidualized and decidualized ESCs. Vertical and horizontal lines show beta-values the position relative to the transcription start site (from − 1500 to + 4000 bp). **c**: Median (50%), 25 and 75% tiles of beta-values of the genes without expression in both types of ESC (9328 genes) are shown in the non-decidualized and decidualized ESCs. **d**: DNA methylation levels (mean ± SD, from − 300 to + 700) of the non-decidualized and decidualized ESCs of up-regulated genes, down-regulated genes, and genes without expression in both types of ESC are shown in boxplots. a, *p* < 0.01 vs. the non-decidualized ESCs of up-regulated genes; **b**, *p* < 0.01 vs. the decidualized ESCs of up-regulated genes; **c**, *p* < 0.01 vs. the non-decidualized ESCs of down-regulated genes; **d**, *p* < 0.01 vs. the decidualized ESCs of down-regulated genes

We examined the DNA methylation statuses around the transcription start sites of differentially expressed genes between the non-decidualized and decidualized ESCs. We obtained 2419 up-regulated and 2276 down-regulated genes in the decidualized ESCs compared with the non-decidualized ESCs from our previous study [5] (Additional file 1: Table S1). In these genes, the DNA methylation statuses from − 1500 to 4000 bp were compared between the ESCs. Among the up-regulated 2419 genes, the regions around the transcription start sites (from − 300 to + 700 bp) were DNA hypomethylated compared to the other regions in both the non-decidualized ESCs (beta-value: 0.096 ± 0.170) and the decidualized ESCs (beta-value: 0.093 ± 0.170) (Fig. 2a). Similarly, among the down-regulated 2276 genes, the regions around the transcription start sites (from − 300 to + 700 bp) were hypomethylated in the both ESC types (beta-values: 0.104 ± 0.183 and 0.101 ± 0.183, respectively) (Fig. 2b). These results indicate that there was no significant difference in the DNA methylation levels around the transcription start sites of the differentially expressed genes between the two ESC types.

Our finding that the DNA hypomethylation around the transcription start sites of the differentially expressed genes is concomitant with the fact that DNA hypomethylations of the promoter regions are associated with gene expression [18, 19]. Therefore, we hypothesized that the genes that were not expressed in either type of ESC were hypermethylated. Therefore, we examined the DNA methylation statuses of these genes (those with FPKM < 0.01). The beta-values of the genes without expression in both ESCs were 0.349 ± 0.339 in non-decidualized ESCs and 0.347 ± 0.341 in decidualized ESCs (Fig. 2c). The genes that were not expressed in either type of ESC were more highly methylated than the differentially expressed genes (Fig. 2d). This suggests that DNA hypomethylation is necessary for differential gene expression between the non-decidualized and decidualized ESCs.

### Differentially methylated genes and their mRNA expression statuses

Of 23 differentially DNA methylated CpG sites, 11 CpG sites were associated with 11 genes (Table 2). Of 11 genes, three genes (transcription factor AP-2 alpha, TFAP2A; ubiquitin conjugating enzyme E2 D2, UBE2D2; component of oligomeric Golgi complex 5, COG5) differed in mRNA expression. TFAP2A was more methylated and less expressed in the decidualized ESCs, while UBE2D2 and COG5 were less methylated and more expressed in the decidualized ESCs. The different DNA methylation statuses of UBE2D2 and COG3 were validated by sodium bisulfite sequencing (Additional file 6: Figure S1).

**Table 2:** Eleven differentially methylated genes between non-decidualized and decidualized ESCs and mRNA expression statuses

Genes (Refseq IDs)	Gene symbols	DNA methylation (beta values)		Expression ratio (Fold change)
		non-decidualized ESCs	decidualized ESCs	decidualized ESCs/non-decidualized ESCs
NM_001032280	TFAP2A	0.001116211	0.7699487	0.602183089
NM_001099284	ZNF239	0.3894365	0.7715948	0.983577659
NM_006897	HOXC9	0	0.3599297	0.957752628
NR_001317	HCG4P6	0.0165225	0.3242724	0.935774769
NM_016078	FAM18B	0.004547609	0.3047889	1.023612243
NM_176810	NLRP13	0.5744109	0.2743416	1
NM_033554	HLA-DPA1	0.6535866	0.3476084	0.915018217
NM_006557	DMRT2	0.8715751	0.5523196	1
NM_181733	COG5	0.7118202	0.3734171	1.801646442
NM_000257	MYH7	0.7474061	0.3890077	1
NM_181838	UBE2D2	0.7447599	0.374194	1.885683062

### DNA methylation profiles of regions with histone modification change during decidualization

**Fig. 3:**
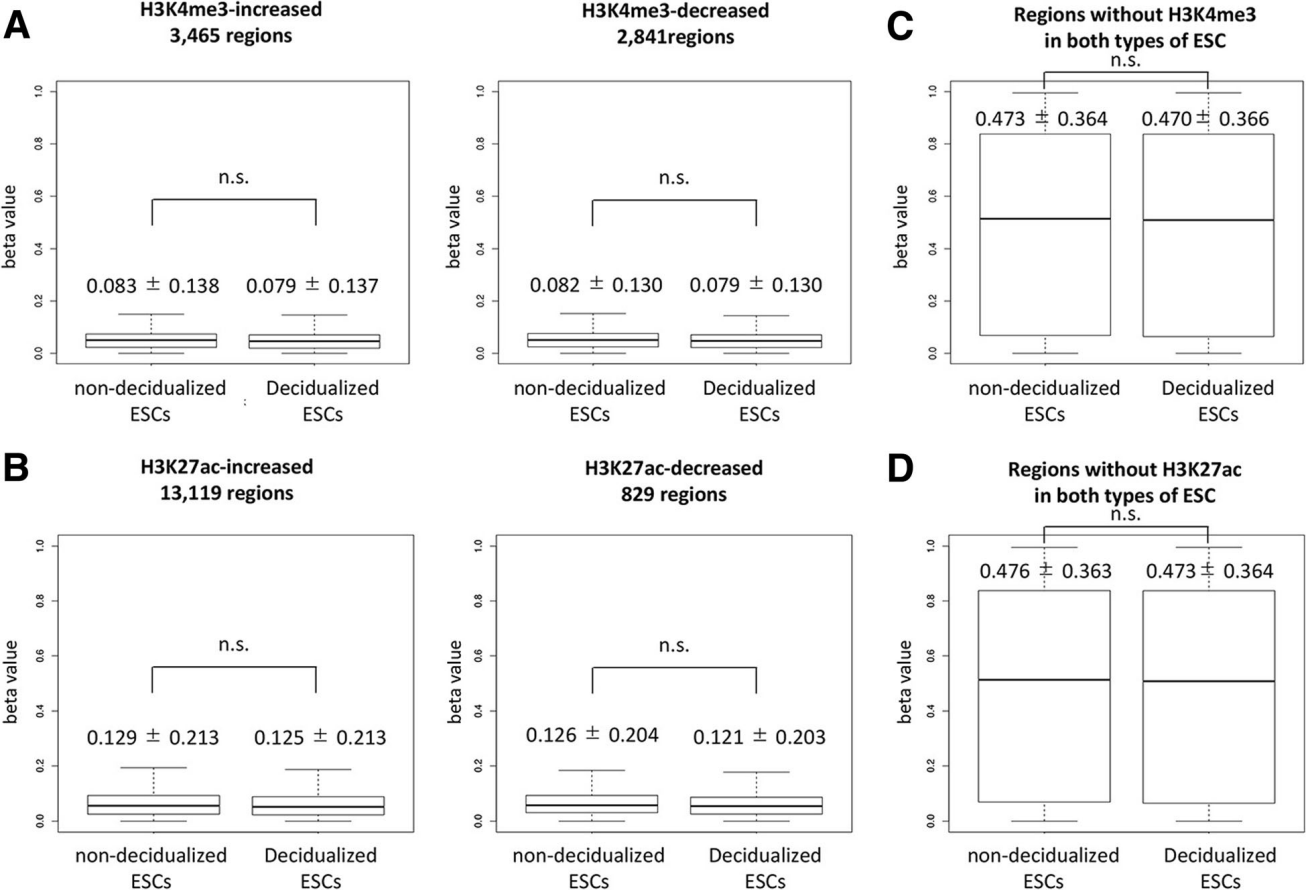
DNA methylation statuses of the differentially histone modified regions between the non-decidualized and decidualized ESCs. **a**: The beta-values of H3K4me3-increased (3465 regions, left) and H3K4me3-decreased (2841 regions, right) are shown in boxplots. Each mean and SD value is shown above the boxplot. **b**: The beta-values of H3K27ac-increased (13,119 regions, left) and H3K27ac-decreased (829 regions, right) are shown in boxplots. **c**: The beta-values of the regions without H3K4me3 in both types of ESC are shown in boxplots. **d**: The beta-values of the regions without H3K27ac in both types of ESC are shown in boxplots. n.s., not significant

Because DNA methylation interacts with other epigenetic features such as histone modifications [15–18], we investigated the DNA methylation statuses in regions in which histone modifications were altered by decidualization according to our previous study [5]. We obtained the regions with increased or decreased histone modifications of H3K4me3 (3465 and 2841 regions, respectively) and H3K27ac (13,119 and 829 regions) during decidualization (Additional file 2: Table S2) [5]. In H3K4me3-increased and -decreased regions, the DNA methylation statuses did not differ significantly between the ESCs (Fig. 3a). In the H3K27ac-increased and -decreased regions, the DNA methylation statuses also did not differ significantly between the ESCs (Fig. 3b). These results also indicate that the regions in which histone modification was altered during decidualization showed DNA hypomethylation status in both types of ESC (Fig. 3a and b). This suggests that DNA hypomethylation is critical for changes in histone modification during decidualization. Therefore, we investigated whether the regions that had no histone modification in either type of ESC were DNA hypermethylated. As shown in Fig. 3c, the regions without H3K4me3 in both types of ESC were more methylated than the regions with different histone modifications between the ESCs (Fig. 3a). Similarly, the regions without H3K27ac in both types of ESC were more methylated (Fig. 3d) than the regions with different histone modifications between the ESCs (Fig. 3b). There was no significant difference in the DNA methylation statuses between the non-decidualized and decidualized ESCs (Fig. 3c and d).

### DNA methylation and histone modification statuses of IGFBP-1 and PRL

*IGFBP-1* and *PRL* are representative markers for decidualization that are upregulated in decidualization [1]. The DNA methylation statuses of the promoter region of *IGFBP-1* and *PRL* were examined by bisulfite sequencing. The *IGFBP-1* promoter region has 27 CpGs between − 501 bp to about + 75 bp and the *PRL* promoter and enhancer regions have 11 CpGs between − 1966 bp to about + 350 bp. In the non-decidualized ESCs, the CpGs in the proximal region of *IGFBP-1* promoter, which exist near the transcriptional factor binding sites (− 263 to + 75 bp), were hypomethylated, whereas the CpGs in the distal region (− 501 to − 442 bp) were methylated (Additional file 7: Figure S2A). The DNA methylation status in the *IGFBP-1* promoter region was not altered by decidualization (Additional file 7: Figure S2A). As for *PRL*, two CpGs (− 4 and − 360 bp) were hypomethylated and the other CpGs were DNA methylated in the non-decidualized ESCs (Additional file 7: Figure S2B). The DNA methylation status in the *PRL* promoter region was not altered by decidualization (Additional file 7: Figure S2B). We next investigated whether the histone modification (H3K27ac and H3K4me3) in the promoter region changes during decidualization under the DNA hypomethylation status. As shown in Additional file 7: Figure S2C, the H3K27ac of *IGFBP-1* showed an increased modification, while that of *PRL* didn’t show a change during decidualization. The H3K4me3 were not altered by decidualization.

### Comparison of the DNA methylation status between ESCs and other cell types

**Fig. 4:**
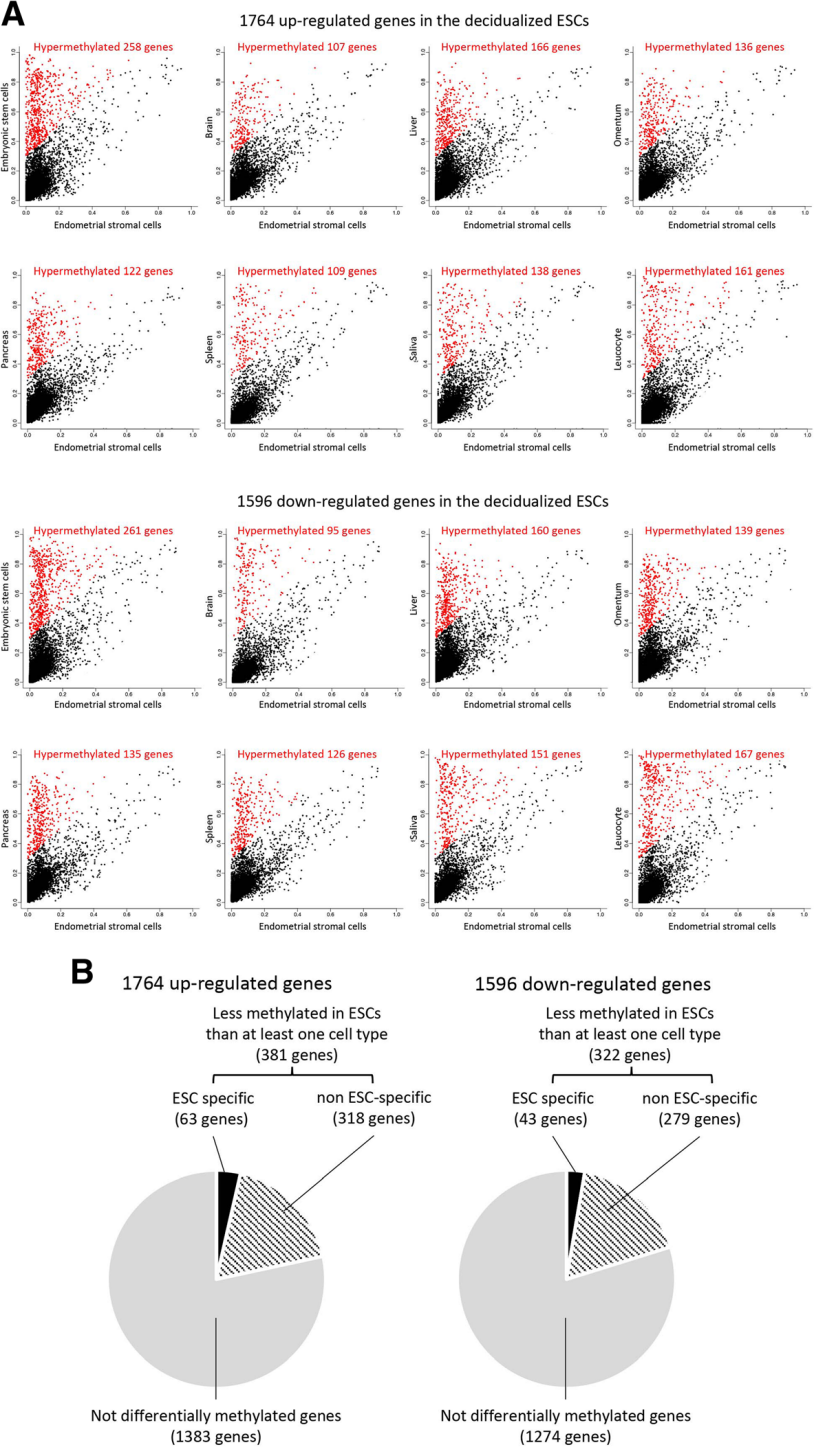
Comparison of the DNA methylation status between the ESCs and other cell types. **a**: Scatterplots of the beta-values in the ESCs and other cell types. The DNA methylation statuses of the genes whose average beta-value from − 300 to + 700 bp in the ESCs was less than 0.2 and whose expression were increased or decreased more than 1.5-fold during decidualization were compared between the ESCs and other cell types (human embryonic stem cells, brain, liver, omentum, pancreas, spleen, saliva and leukocyte). The average beta-values from − 300 to + 700 bp are plotted according to the beta-values of the ESCs (horizontal axis) and other cell types (vertical axis). Dashed lines show minimum beta-value changes of 0.30. Red dots indicate the genes that are differentially DNA hypermethylated (Δbeta-values > 0.3) genes in other cell types compared to the ESCs. **b**: Up-regulated and down-regulated genes are classified into 3 groups based on the specificity of the DNA hypomethylation in the ESCs. ESC-specific genes indicate the genes that are less methylated in the ESCs than any other cell types. Non-ESC-specific genes indicate the genes in which one or more cell types show DNA hypomethylation similar to the ESCs

To investigate whether DNA hypomethylated genes in ESCs with differential expression before and after decidualization exhibit DNA hypermethylation statuses in cell types other than ESCs, we examined the DNA methylation statuses of these genes in publicly available Illumina HumanMethylation450 data of embryonic stem cells, brain, liver, omentum, pancreas, spleen, saliva and leucocyte. We first extracted the genes whose average beta-value from − 300 to + 700 bp in the ESCs was < 0.2 and whose expression were up-regulated or down-regulated during decidualization (Additional file 4: Table S4). As a result, 1764 up-regulated and 1596 down-regulated genes were obtained. In 1764 up-regulated genes, a number of genes (107 to 258 genes) were differentially DNA hypermethylated in other cell types compared to the ESCs (Δbeta-values > 0.3, Fig. 4a). Similarly, in 1596 down-regulated genes, a number of genes (95 to 261 genes) were DNA hypermethylated in other cell types (Fig. 4a). We next investigated the genes that are specifically DNA hypomethylated in ESCs. Of the 1764 up-regulated genes, 381 genes were less methylated in the ESCs than in at least one cell type and 63 genes showed ESC-specific DNA hypomethylation (Fig. 4b and Additional file 4: Table S4). In 1596 down-regulated genes, 322 genes were less methylated in the ESCs than in at least one cell type and 43 genes showed ESC-specific DNA hypomethylation (Fig. 4b and Additional file 4: Table S4). In 106 (63 + 43) of ESC-specific hypomethylated genes, Gene Ontology (GO) analysis showed that these genes included metalloproteinase and collagen genes which compose extracellular matrix and interstitial matrix (Additional file 5: Table S5).

### The expression statuses of the ESC-specific hypomethylated genes in other cell types

We next evaluated the ESC-specific DNA hypomethylation was linked to ESC-specific gene expression. We examined the expression statuses of the ESC-specific hypomethylated and differentially expressed genes during decidualization (63 up-regulated and 43 down-regulated genes) in publicly available RNA sequencing data of embryonic stem cells, brain, liver, pancreas, spleen and saliva. Then the expression levels were compared between the ESCs and other cell types. The ESC-specific hypomethylated and up-regulated genes showed significantly lower expression statuses in brain, liver, pancreas, spleen and saliva than the decidualized ESCs (*p* < 0.01, Additional file 9: Figure S4). The ESC-specific hypomethylated and down-regulated genes showed significantly lower expression statuses in other cell types than the non-decidualized ESCs (*p* < 0.05, Additional file 9: Figure S4). These results indicate that the differentially expressed genes during decidualization with ESC-specific hypomethylation were kept down-regulated in other cell types.

## Discussion

Genome-wide DNA methylation analysis revealed no remarkable difference in DNA methylation profiles between the non-decidualized and decidualized ESCs. Only 0.0048% (Δbeta-value > 0.3) of the examined CpG sites were differentially methylated between the two types of ESC. DNA methylation is specific to each cell/tissue type [7], and is involved in cell/tissue-specific gene expression [13, 14]. During cell differentiation, DNA methylation changes occur early in cell lineage commitment, and only a few changes arise during the terminal differentiation [20]. Transcriptional repression of genes by DNA methylation is essentially limited to specific genes that need to be permanently silenced. These observations suggest that once cells are terminally differentiated, the DNA methylation status is stable and does not change easily [21]. Therefore, it is not surprising that DNA methylation did not change in the ESCs during decidualization. Decidualization would be a cellular differentiation of ESCs to another cell type only with gene expression changes.

The DNA methylation statuses around the gene promoter and within the gene body are closely associated with mRNA expression [18, 19]. Most of the 2419 up-regulated and 2276 down-regulated genes didn’t show any difference in the DNA methylation statuses between the two types of ESC. This result indicates that DNA methylation is not directly associated with the change of gene expression during decidualization. Notably, the present study showed that the DNA methylation statuses of the promoter regions of the genes with different expression between the two types of ESCs were hypomethylated, while those of the genes that are not expressed in either type of ESC were hypermethylated. This is concomitant with the general finding that DNA hypomethylation around the transcription start sites is associated with gene expression [7]. Furthermore, this finding suggests that the DNA hypomethylation of the gene promoter region underlies the variability of the gene expression during decidualization, and the expression of the genes with DNA hypomethylation at the promoter region is regulated by other mechanisms such as histone modifications and transcription factor binding [4, 22].

Our previous study showed that histone modifications were genome-widely altered in the ESCs during decidualization [5]. Since histone modifications interact with DNA methylation each other, we investigated in the present study whether the DNA methylation statuses were also altered in the regions in which histone modifications were altered during decidualization. The results showed that there was no difference in the DNA methylation statuses of those regions between the non-decidualized and decidualized ESCs. This is not surprising because histone modifications are involved in short-term and variable epigenetic regulation, while DNA methylation does not play a role in such fine-tuning of gene expression [23]. Notably, the regions in which histone modifications were altered during decidualization showed DNA hypomethylation statuses compared with the regions without the histone modification changes in the both types of ESC. Active histone modifications under the DNA hypomethylation status induce loosened chromatin structures, which promote the access of transcription factors to the gene promoter regions to induce gene expression [24, 25]. In the present study, we found that DNA hypomethylation underlies the change of histone acetylation status during decidualization in the promoter region of *IGFBP-1*. We previously showed that the change of histone acetylation status was associated with the induction of the *IGFBP-1* by making the promoter regions accessible to transcription factors during decidualization [26]. Taken together, these observations suggest that, during decidualization, DNA hypomethylation of the promoter regions is maintained for the change of the histone modifications and the subsequent transcription factor binding.

Houshdaran et al. examined the DNA methylation statuses of the endometrium at the proliferative and mid-secretory phases by a genome-wide approach using Infinium HumanMethylation27K BeadChip [27]. They reported that 66 of 27,578 CpGs (0.2923%) were differentially methylated between the two phases. There seems to be a discrepancy between their result and our result (0.0048%). They used the cut-off of beta-values as > 0.136 for extracting differentially methylated CpG sites, while we used the cut-off values as > 0.30, which is generally accepted. Furthermore, other possible explanations for the discrepancy may lie in the different platforms of microarray, races, and study designs (in vivo and in vitro studies). Especially, since the samples in their in vivo study include other small structures such as capillaries and blood cells, the difference may have been overestimated.

By the comparison of DNA methylation pattern between the ESCs and other cell types, even the decidualized ESC-specific genes were already DNA hypomethylated in the non-decidualized ESCs, while other cell types showed DNA hypermethylation of the same genes. Furthermore, the ESC-specific DNA hypomethylation was linked to ESC-specific gene expression. The expressions of these genes only after decidualization should be caused by binding of appropriate transcription factors that would be coupled with histone acetylation and were important for the structural component of the decidualized endometrium. This suggests that the ESC-specific DNA hypomethylation profile defines the potential of differentiation in ESCs and contributes to decidualization. Taken together, ESCs are likely a terminally differentiated cell type, and decidualization would not be a differentiation process but simply a reaction to certain stimuli such as steroid hormones that result in morphological and gene expression changes. Notably, not all of the genes were specifically hypomethylated in the ESCs. However, since transcriptional repression of genes by DNA methylation is limited to specific genes with permanent silencing, it is not surprising that not all genes that were differentially expressed during decidualization showed ESC-specific DNA hypomethylation.

## Conclusion

The present study showed that culturing ESCs with estrogen/progesterone does not distort the physiological pattern of DNA methylation, although histone modifications and mRNA expression dynamically change. In terms of the DNA methylation profile, ESCs before decidualization may have already been established as a fixed cell type. Taken our previous study on histone modifications into consideration [5], our results suggest that histone modifications rather than DNA methylation may have a preferential role in the regulation of gene expression during decidualization in ESCs, and that the regulation by histone modifications is supported by stable DNA hypomethylation.

## Methods

### ESC isolation and cell culture

ESCs were isolated and cultured as previously reported [5, 28, 29]. Human endometrial tissues were obtained from patients (aged 41–44 years) who underwent hysterectomy for myoma uteri or early stage of cervical cancer. They had a normal menstrual cycle. All endometrial samples were diagnosed as being in the late proliferative phase according to published criteria by histologic examination [30]. Tissue samples were washed with Phenol Red-free DMEM which contained 4 mM glutamine, 50 μg/mL streptomycin, and 50 IU/mL penicillin, and minced into pieces of less than 1 mm^3^. ESCs were isolated as previously reported [31]. After the minced tissues underwent enzymatic digestion with 0.2% collagenase in a shaking water bath for 2 h at 37 °C, endometrial stromal cells were isolated by filtration through a 70-μm nylon mesh. The filtrates were washed with the medium three times. The separated endometrial stromal cells were seeded at 10^5^ cells/cm^2^ in 75-cm^2^ tissue culture flasks and incubated in Phenol Red-free DMEM which contained glutamine, antibiotics, and 10% dextran-coated charcoal-stripped fetal bovine serum at 37 °C, 95% air and 5% CO2. At confluence, cells were resuspended with 1 x trypsin-EDTA and subcultured into 75-cm^2^ tissue culture flasks. When cells were harvested at 80% confluence after the first passage, ESCs were incubated with or without E (10^− 8^ mol/L) and MPA (10^− 6^ mol/L) for 14 days at 37 °C, 95% air, and 5% CO_2_ to induce decidualization as previously reported [32]. The medium was changed every other day. Decidualization was confirmed by the induction of mRNA expression of *IGFBP-1* and *PRL*, which are specific markers of decidualization, using the primers shown in Additional file 3: Table S3 [1, 29, 33]. The results were shown in Additional file 8: Figure S3. The ESCs were incubated and used for DNA methylation experiments.

### Illumina Infinium HumanMethylation450 BeadChip assay

Genomic DNA was isolated from the culture ESCs using a Qiagen Genomic DNA kit (Qiagen, Valencia, CA, USA). DNA methylation was analyzed with an Illumina Infinium assay with the HumanMethylation450 BeadChip (Illumina, San Diego, CA, USA), which interrogates a total of 482,421 CpGs spread across the distal promoter regions of transcription start sites to 3′-UTR of consensus coding sequences. Methylated and unmethylated signals were used to compute beta-values, which are quantitative scores of the DNA methylation levels, ranging from 0 (completely unmethylated) to 1 (completely methylated). The BeadChip was scanned on a BeadArray Reader (Illumina) according to the manufacturer’s instructions. CpGs with “detection *p* values” > 0.05 (computed from the background based on negative controls) and CpGs on Y chromosome were eliminated from further analysis, leaving 482,005 CpGs valid for use. CpGs whose methylation status differed between non-decidualized ESCs and decidualized ESCs were identified by the difference of beta-values. CpGs with beta values greater than 0.3 were extracted as differentially methylated CpGs. The raw data was deposited in the Gene Expression Omnibus (number GSE108143). We used NCBI Reference Sequence Database (https://www.ncbi.nlm.nih.gov/refseq/) as reference genes.

### Differentially expressed genes between non-decidualized ESCs and decidualized ESCs

Genome-wide mRNA expression data of two pairs of non-decidualized ESCs and decidualized ESCs were obtained from our previous RNA sequencing study [5]. RNA-seq raw data was deposited in the Gene Expression Omnibus (number GSE57010). Gene expression was mapped and quantified by TopHat [34], and cufflinks [35]. Gene expression values were calculated as fragments per kilobase of exon unit per million mapped reads (FPKM). The mean values in decidualized and non-decidualized ESCs were calculated, respectively. We defined the genes whose FPKM values increased or decreased more than 1.5-fold in decidualization as up-regulated or down-regulated genes during decidualization, respectively.

### Regions with different histone modification statuses between non-decidualized ESCs and decidualized ESCs

The genome-wide statuses of the histone modifications of H3K4me3 and H3K27ac in decidualized and non-decidualized ESCs were obtained from our previous study of chromatin immuno-precipitation (ChIP) sequencing [5]. ChIP-seq raw data was deposited in the Gene Expression Omnibus (number GSE57010). Regions with different histone modifications were mapped and identified by Bowtie [36], followed by Model-based Analysis of ChIP-Seq (MACS) [37]. The regions with different histone modifications that were shared by the two individuals in the study were considered as the regions in which histone modification signals were altered by decidualization.

### Comparison of the DNA methylation status between ESCs and other cell types

We obtained the DNA methylation data of 8 cell types other than ESCs examined by Illumina Infinium HumanMethylation450 BeadChip from gene expression omnibus database (human embryonic stem cells, brain and leukocyte from GSE52576; spleen, liver, omentum, pancreas and saliva from GSE 48472). Genes whose average beta-value from − 300 to + 700 bp in the ESCs was less than 0.2 and whose expression were increased or decreased more than 1.5-fold during decidualization were extracted. Then, the average beta-values from − 300 to + 700 bp of the genes in each cell type were plotted in comparison with the ESCs. To investigate the genes that are specifically DNA hypomethylated in the ESCs, we extracted the genes that were DNA hypomethylated in the ESCs compared with any other cell types (Δbeta-values > 0.3).

### The expression statuses of the ESC-specific hypomethylated genes in other cell types

The RNA sequencing of brain, liver, pancreas, spleen, saliva was retrieved from the GTEx portal (https://storage.googleapis.com/gtex_analysis_v7/rna_seq_data/GTEx_Analysis_2016-01-15_v7_RNASeQCv1.1.8_gene_reads.gct.gz). These expression data were scaled together with the expression data of the ESCs in R [38], and z-score was obtained. Of the ESC-specific hypomethylated and differentially expressed genes during decidualization (up-regulated 63 genes and down-regulated 43 genes), 51 and 35 genes were subjected to the analysis, respectively. The expression levels of these genes were compared between the ESCs (non-decidualized ESCs and decidualized ESC) and other cell types (brain, liver, pancreas, spleen and saliva). The expression statuses of these genes in each cell type were plotted.

### Sodium bisulfite sequencing

Using the same DNA samples used in the BeadChip array, bisulfite reactions were performed using an EpiTect Bisulfite kit (Qiagen) with the following conditions: 95 °C for 5 min, 65 °C for 85 min, 95 °C for 5 min, and 65 °C for 175 min as previously reported [14, 39, 40]. The bisulfite converted DNA was amplified by PCR using the primer pairs shown in Additional file 3: Table S3 under the thermocycling conditions (95 °C for 10 min, and 40 cycles of 94 °C for 30 s, 57 °C for 30 s, and 72 °C for 1 min followed by 10 min of final extension at 72 °C). The resulting products were cloned into pGEM-T easy vector (Promega, Tokyo, Japan). After sequencing reaction using a BigDye Terminator V3.1 kit (Applied Biosystems, Foster City, CA, USA), sequencing was performed with a 3130xl Genetic Analyzer (Applied Biosystems) as previously reported [24, 41]. QUMA (http://quma.cdb.riken.jp/) was used to analyze the bisulfite sequencing data [42]. The bisulfite PCR primers are shown in Additional file 3: Table S3.

### Statistical analysis and bioinformatics

The significance of difference between two groups was determined with an unpaired *t* test. The significance of difference between multiple groups was determined with pairwise comparisons with *p*-value adjustment by Holm method. Statistical analyses were performed using SPSS for Windows version 11 (SPSS, Inc). Differences with *P* < 0.01 or *P* < 0.05 were considered significant. CpGs included in the upstream region, gene body region and downstream regions of genes, and the histone modified regions, were detected with Bedtools [43]. DAVID Bioinformatics Resources v. 6.7 was used to determine whether the functional annotation of the differentially expressed genes under ESC-specific DNA hypomethylation was enriched for specific GO terms [44]. In the GO analyses, *P* < 0.01 were considered to indicate significant enrichment.

## Additional files

Additional file 1: **Table S1.** Differentially expressed genes between the non-decidualized and decidualized ESCs and the number of the CpG sites around the transcription start sites (− 1500 to 4000 bp) of the genes. (XLSX 9 kb)

Additional file 2: **Table S2.** Regions with different histone modifications between the non-decidualized and decidualized ESCs. (XLSX 10 kb)

Additional file 3: **Table S3.** Primer pairs used in real-time RT-PCR and sodium bisulfite sequencing. (XLSX 10 kb)

Additional file 4: **Table S4.** ESC-hypomethylated genes (average beta-value from − 300 to + 700 bp is < 0.2) and their methylation levels in other cell types. (XLSX 32504 kb)

Additional file 5: **Table S5.** Differentially expressed genes (106 genes) during decidualization under ESC-specific DNA hypomethylation. (XLSX 10 kb)

Additional file 6: **Figure S1.** DNA methylation status of the CpGs in *UBE2D2* and *COG5*. A; DNA methylation statuses of the CpGs between + 4527 and + 4784 of *UBE2D2* were analyzed in the non-decidualized and decidualized ESCs. The CpG of + 4527 was detected as differentially methylated CpG in Illumina HumanMethylation45K analysis. B; DNA methylation statuses of the CpGs between − 433 to − 194 of *COG5* were analyzed in the non-decidualized and decidualized ESCs. The CpG of − 379 was detected as differentially methylated CpG in Illumina HumanMethylation450K analysis. The diagrams show the distribution of CpGs. The position of the transcription start site is designated as + 1. Open and filled circles indicate unmethylated and methylated CpGs status, respectively. (JPG 379 kb)

Additional file 7: **Figure S2.** DNA methylation and histone modification statuses in the *IGFBP-1* and *PRL* promoter regions. A and B; The DNA methylation statuses of CpGs in the promoter regions of *IGFBP-1* (A) and *PRL* (B). A; Methylation status of all the CpGs between − 501 and + 75 (27 CpGs) was analyzed in the non-decidualized and decidualized ESCs. B; DNA methylation status of all the CpGs between − 1966 and + 350 (11 CpGs) was analyzed in the non-decidualized and decidualized ESCs. The diagrams show the distribution of CpGs. The position of the transcription start site is designated as + 1. Open and filled circles indicate unmethylated and methylated CpGs status, respectively. C and D; H3K27ac and H3K4me3 statuses in the promoter regions of *IGFBP-1* (C) and *PRL* (D). Peak call regions detected by the analysis using MACS are shaded. (JPG 392 kb)

Additional file 8: **Figure S3.** mRNA expression status of *IGFBP-1* and *PRL.* mRNA expression of *IGFBP-1* and *PRL* were analyzed by qRT-PCR using primers shown in Additional file: Table S3. *GAPDH* was used as an internal control. The value of mRNA was normalized to that of the internal control (*GAPDH*). (JPG 265 kb)

Additional file 9: **Figure S4.** Comparison of the expression status of the ESC-specific hypomethylated genes between the ESCs and other cell types. A and B; The mRNA expression statuses of ESC-specific hypomethylated and up-regulated (A) or down-regulated (B) genes. The expression levels of each gene in 7 cell types are indicated as dots (left column) and boxplots (right column). The significance of difference between multiple groups was determined with pairwise comparisons with *p*-value adjustment by Holm method. ESC, non-decidualized ESCs; dESC, decidualized ESCs. (JPG 788 kb)

## Abbreviations

ChIP: chromatin immuno-precipitation
CO2: carbon dioxide
COG5: component of oligomeric Golgi complex 5
CpG: Cytosine-phosphate-Guanine
DAVID: Database for Annotation, Visualization and Integrated Discovery
DMEM: Dulbecco’s Modified Eagle’s Medium
DNA: Deoxyribonucleic acid
E: estrogen
EDTA: ethylenediaminetetraacetic acid
ESC: endometrial stromal cell
FPKM: fragments per kilobase of exon unit per million mapped reads
GO: gene ontology
H3K27ac: histone H3 lysine 27 acethylation
H3K4me3: histone H3 lysine 4 tri-methylation
IGFBP-1: Insulin-like growth factor-binding protein 1
MACS: Model-based Analysis of ChIP-Seq
MPA: medroxyprogesterone acetate
mRNA: messenger ribonucleic acid
PCR: polymerase chain reaction
PRL: prolactin
QUMA: quantification tool for methylation analysis
SPSS: Statistical Package for Social Science
TFAP2A: transcription factor AP-2 alpha
UBE2D2: ubiquitin conjugating enzyme E2 D2
UTR: untranslated region
