# Correction: Genome-wide DNA methylation analysis revealed stable DNA methylation status during decidualization in human endometrial stromal cells

**DOI:** 10.1186/s12864-024-10222-4

**Published:** 2024-04-05

**Authors:** Ryo Maekawa, Isao Tamura, Masahiro Shinagawa, Yumiko Mihara, Shun Sato, Maki Okada, Toshiaki Taketani, Hiroshi Tamura, Norihiro Sugino

**Affiliations:** Department of Obstetrics and Gynecology, Yamaguchi University Graduate School of Medicine, Minami Kogushi 1-1-1, 755-8505 Ube, Japan

**Correction**: *BMC Genomics***20**, 324 (2019). 10.1186/s12864-019-5695-0

Following publication of the original article it was reported that Additional files 6–8 contained the wrong files, namely duplicates of the figures appearing in the article body. The correct files are included with in this Correction.

### Electronic supplementary material

Below is the link to the electronic supplementary material.

Supplementary Material 1

Supplementary Material 2

Supplementary Material 3

Supplementary Material 4

