# Supplementary figures and images for: Genome-wide DNA methylation analysis revealed stable DNA methylation status during decidualization in human endometrial stromal cells
Source: BMC Genomics. 2019 Apr 29;20:324. doi: 10.1186/s12864-019-5695-0 (PMC6489213; doi:10.1186/s12864-019-5695-0)

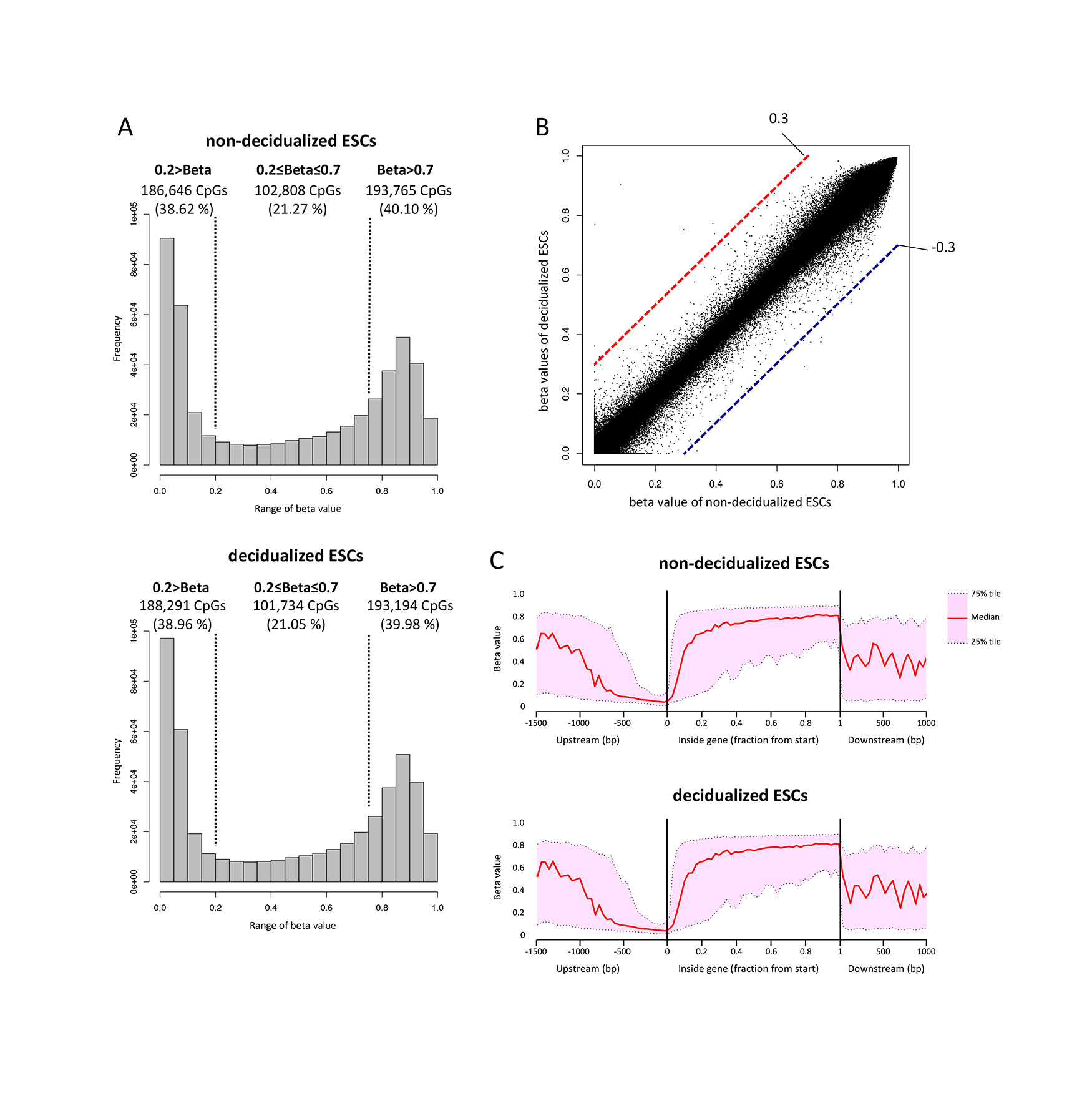

Supplement: Supplementary file 6 — Figure S1. DNA methylation status of the CpGs in UBE2D2 and COG5. A; DNA methylation statuses of the CpGs between + 4527 and + 4784 of UBE2D2 were analyzed in the non-decidualized and decidualized ESCs. The CpG of + 4527 was detected as differentially methylated CpG in Illumina HumanMethylation45K analysis. B; DNA methylation statuses of the CpGs between − 433 to − 194 of COG5 were analyzed in the non-decidualized and decidualized ESCs. The CpG of − 379 was detected as differentially methylated CpG in Illumina HumanMethylation450K analysis. The diagrams show the distribution of CpGs. The position of the transcription start site is designated as + 1. Open and filled circles indicate unmethylated and methylated CpGs status, respectively. (JPG 379 kb) [file 12864_2019_5695_MOESM6_ESM.jpg]

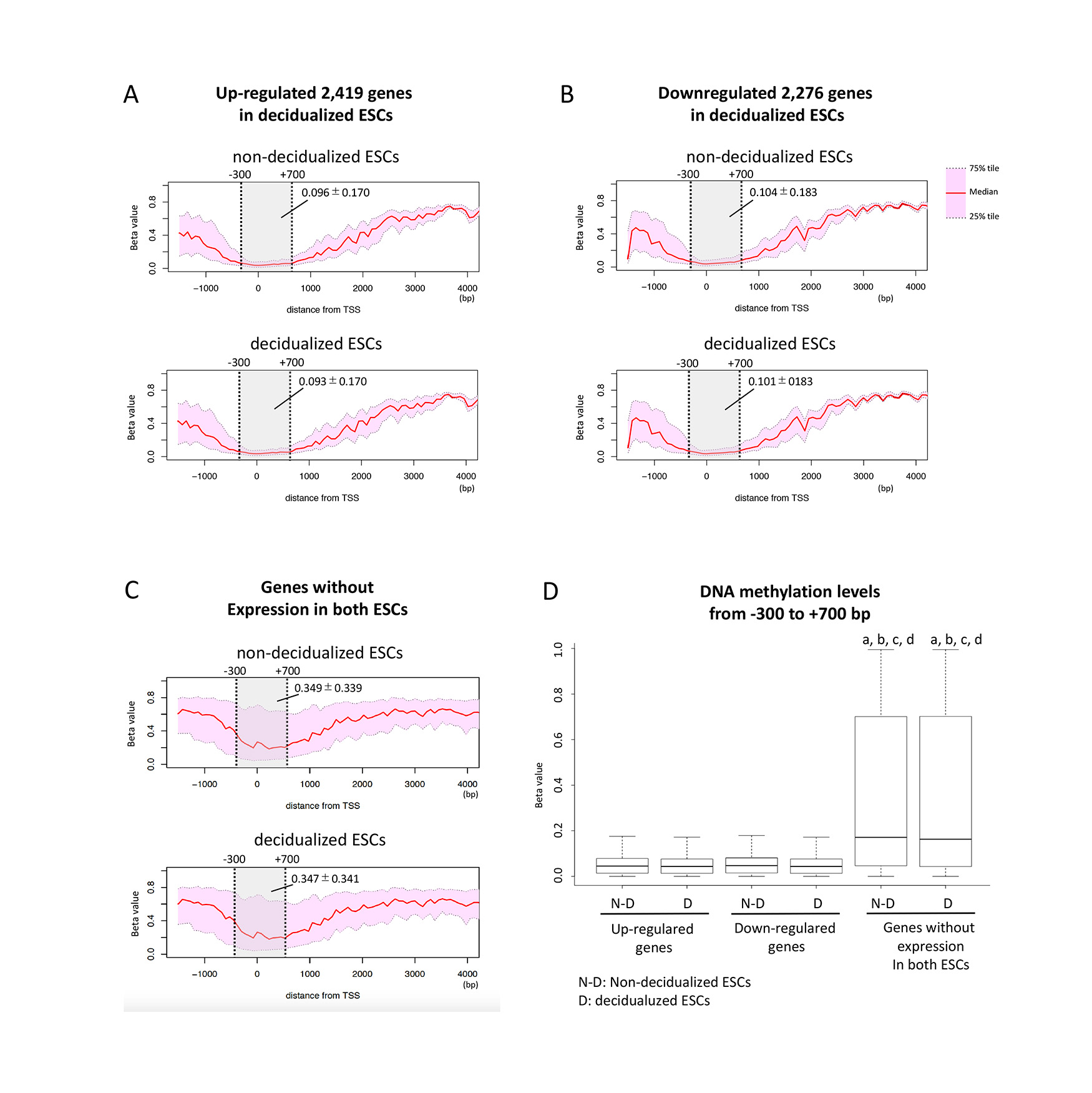

Supplement: Supplementary file 7 — Figure S2. DNA methylation and histone modification statuses in the IGFBP-1 and PRL promoter regions. A and B; The DNA methylation statuses of CpGs in the promoter regions of IGFBP-1 (A) and PRL (B). A; Methylation status of all the CpGs between − 501 and + 75 (27 CpGs) was analyzed in the non-decidualized and decidualized ESCs. B; DNA methylation status of all the CpGs between − 1966 and + 350 (11 CpGs) was analyzed in the non-decidualized and decidualized ESCs. The diagrams show the distribution of CpGs. The position of the transcription start site is designated as + 1. Open and filled circles indicate unmethylated and methylated CpGs status, respectively. C and D; H3K27ac and H3K4me3 statuses in the promoter regions of IGFBP-1 (C) and PRL (D). Peak call regions detected by the analysis using MACS are shaded. (JPG 392 kb) [file 12864_2019_5695_MOESM7_ESM.jpg]

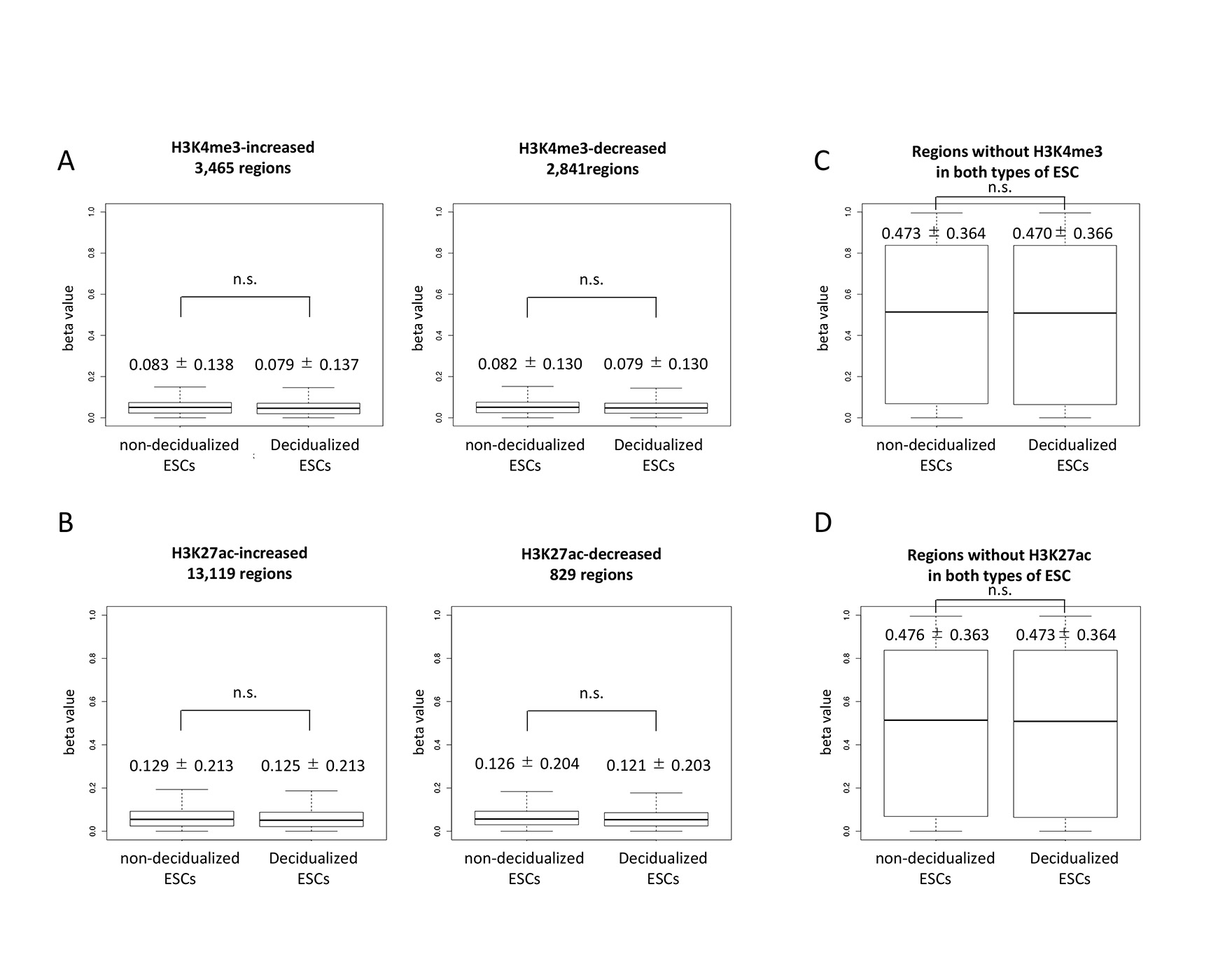

Supplement: Supplementary file 8 — Figure S3. mRNA expression status of IGFBP-1 and PRL. mRNA expression of IGFBP-1 and PRL were analyzed by qRT-PCR using primers shown in Additional file: Table S3. GAPDH was used as an internal control. The value of mRNA was normalized to that of the internal control (GAPDH). (JPG 265 kb) [file 12864_2019_5695_MOESM8_ESM.jpg]

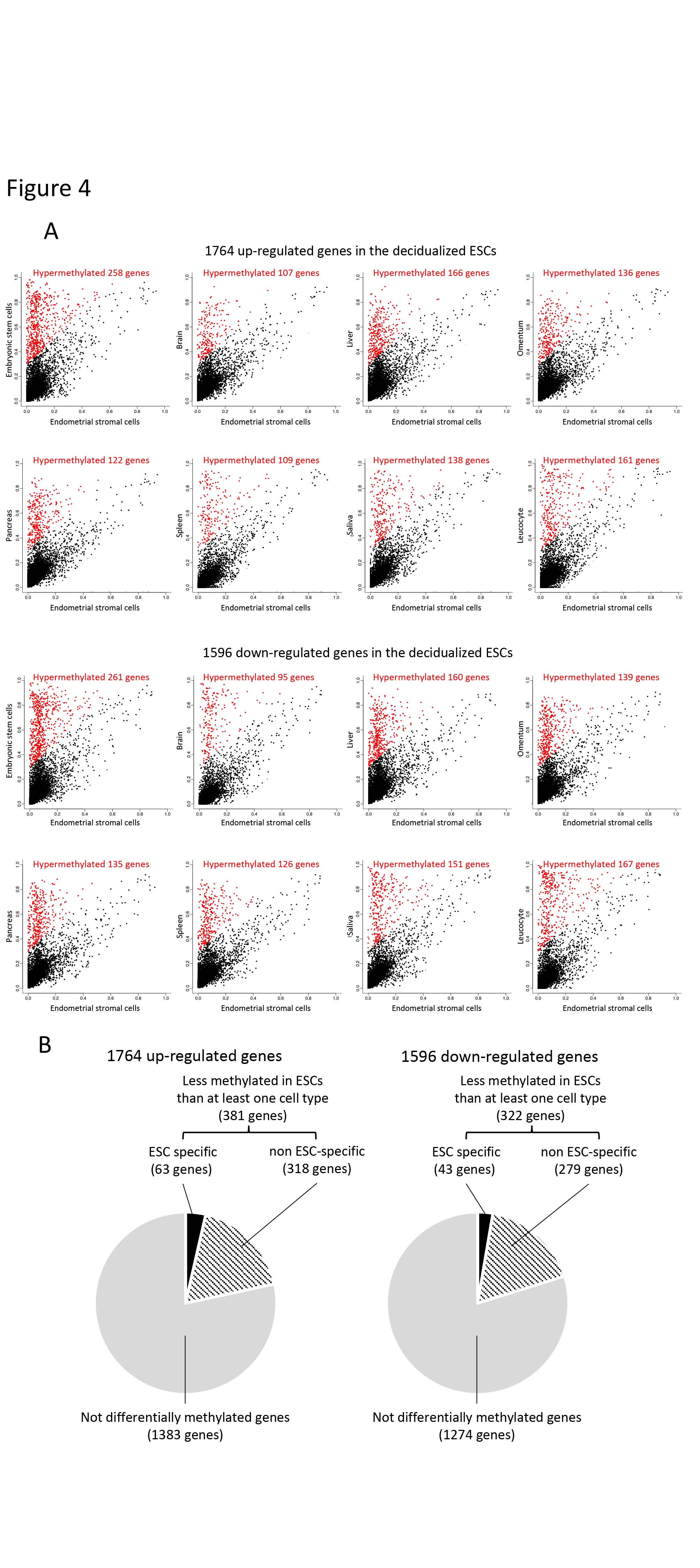

Supplement: Supplementary file 9 — Figure S4. Comparison of the expression status of the ESC-specific hypomethylated genes between the ESCs and other cell types. A and B; The mRNA expression statuses of ESC-specific hypomethylated and up-regulated (A) or down-regulated (B) genes. The expression levels of each gene in 7 cell types are indicated as dots (left column) and boxplots (right column). The significance of difference between multiple groups was determined with pairwise comparisons with p-value adjustment by Holm method. ESC, non-decidualized ESCs; dESC, decidualized ESCs. (JPG 788 kb) [file 12864_2019_5695_MOESM9_ESM.jpg]
