# Supplementary material for: Correction: Genome-wide DNA methylation analysis revealed stable DNA methylation status during decidualization in human endometrial stromal cells
Source: BMC Genomics. 2024 Apr 5;25:343. doi: 10.1186/s12864-024-10222-4 (PMC10996215; doi:10.1186/s12864-024-10222-4)
Supplement: Supplementary file 1 — Supplementary Material 1 [file 12864_2024_10222_MOESM1_ESM.pptx]

## Slide 1
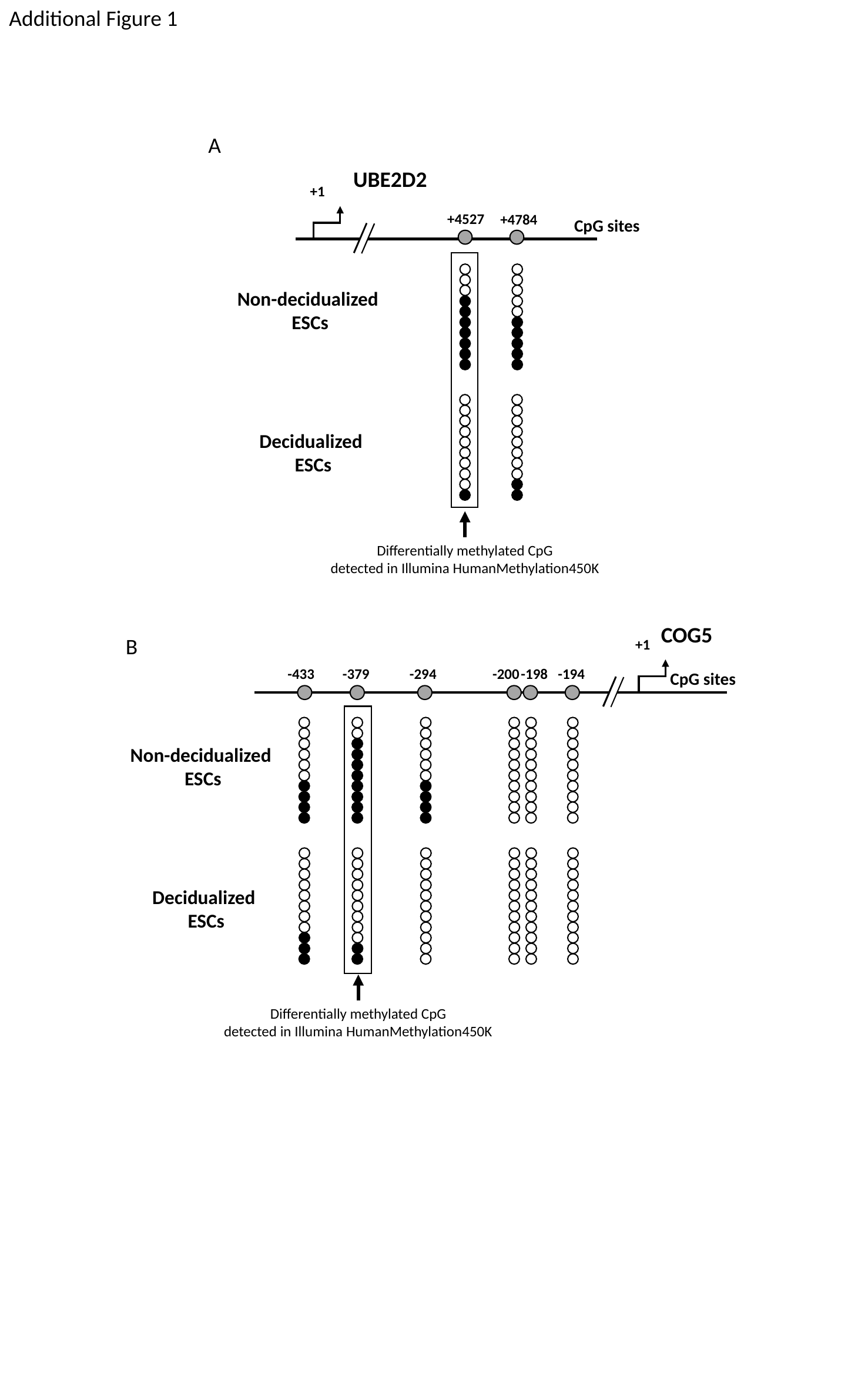

Additional Figure 1
A
UBE2D2
+1
+4527
+4784
CpG sites
Non-decidualized
ESCs
Decidualized
ESCs
Differentially methylated CpG
detected in Illumina HumanMethylation450K
COG5
B
+1
-433
-379
-294
-200
-198
-194
CpG sites
Non-decidualized
ESCs
Decidualized
ESCs
Differentially methylated CpG
detected in Illumina HumanMethylation450K
