# Supplementary material for: Correction: Genome-wide DNA methylation analysis revealed stable DNA methylation status during decidualization in human endometrial stromal cells
Source: BMC Genomics. 2024 Apr 5;25:343. doi: 10.1186/s12864-024-10222-4 (PMC10996215; doi:10.1186/s12864-024-10222-4)
Supplement: Supplementary file 2 — Supplementary Material 2 [file 12864_2024_10222_MOESM2_ESM.pptx]

## Slide 1
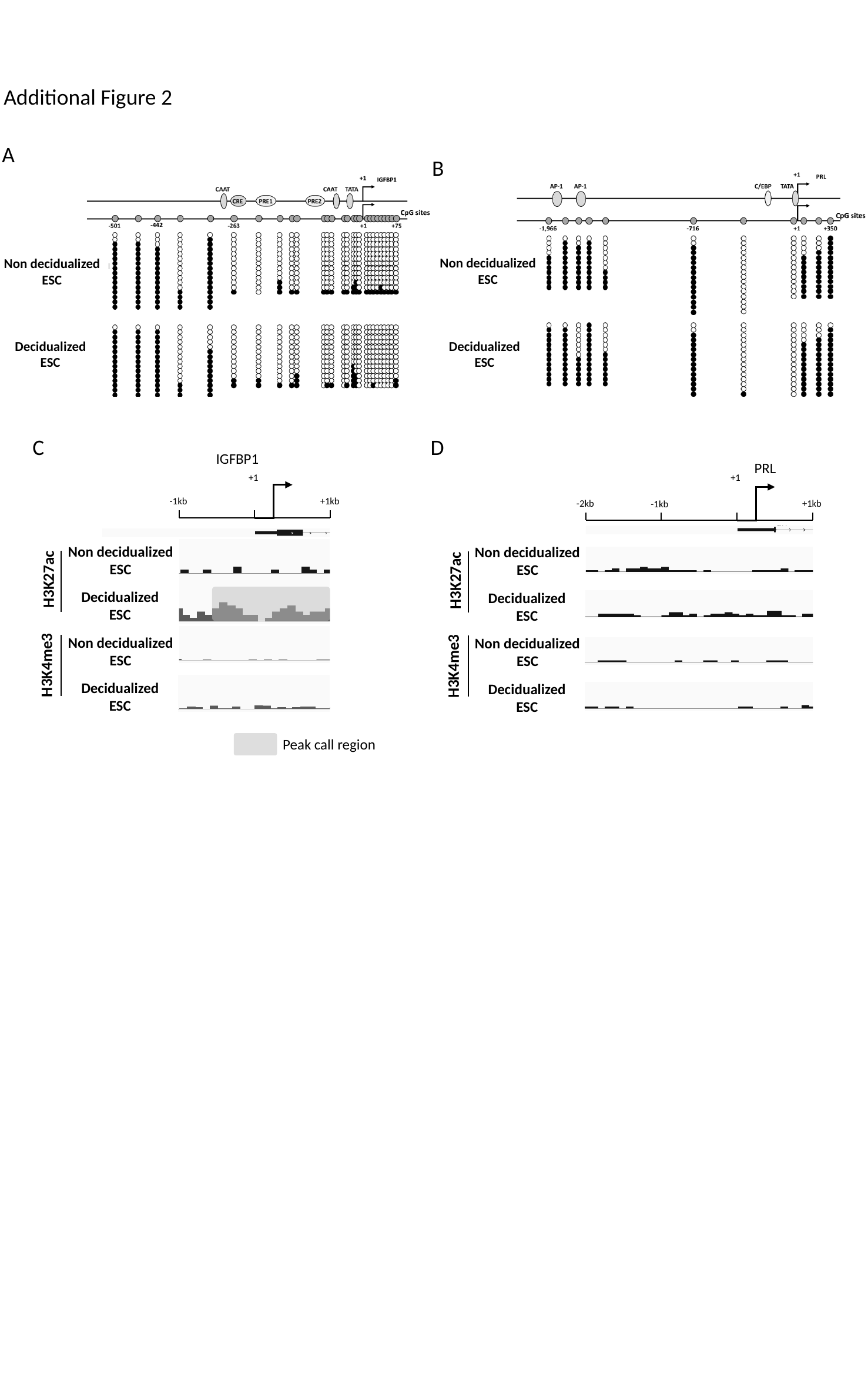

Additional Figure 2
A
B
Non decidualized
ESC
Non decidualized
ESC
Decidualized
ESC
Decidualized
ESC
C
D
IGFBP1
PRL
+1
+1
-1kb
+1kb
-2kb
+1kb
-1kb
Non decidualized
ESC
Non decidualized
ESC
H3K27ac
H3K27ac
Decidualized
ESC
Decidualized
ESC
Non decidualized
ESC
Non decidualized
ESC
H3K4me3
H3K4me3
Decidualized
ESC
Decidualized
ESC
Peak call region
