# Supplementary material for: Correction: Genome-wide DNA methylation analysis revealed stable DNA methylation status during decidualization in human endometrial stromal cells
Source: BMC Genomics. 2024 Apr 5;25:343. doi: 10.1186/s12864-024-10222-4 (PMC10996215; doi:10.1186/s12864-024-10222-4)
Supplement: Supplementary file 3 — Supplementary Material 3 [file 12864_2024_10222_MOESM3_ESM.pptx]

## Slide 1
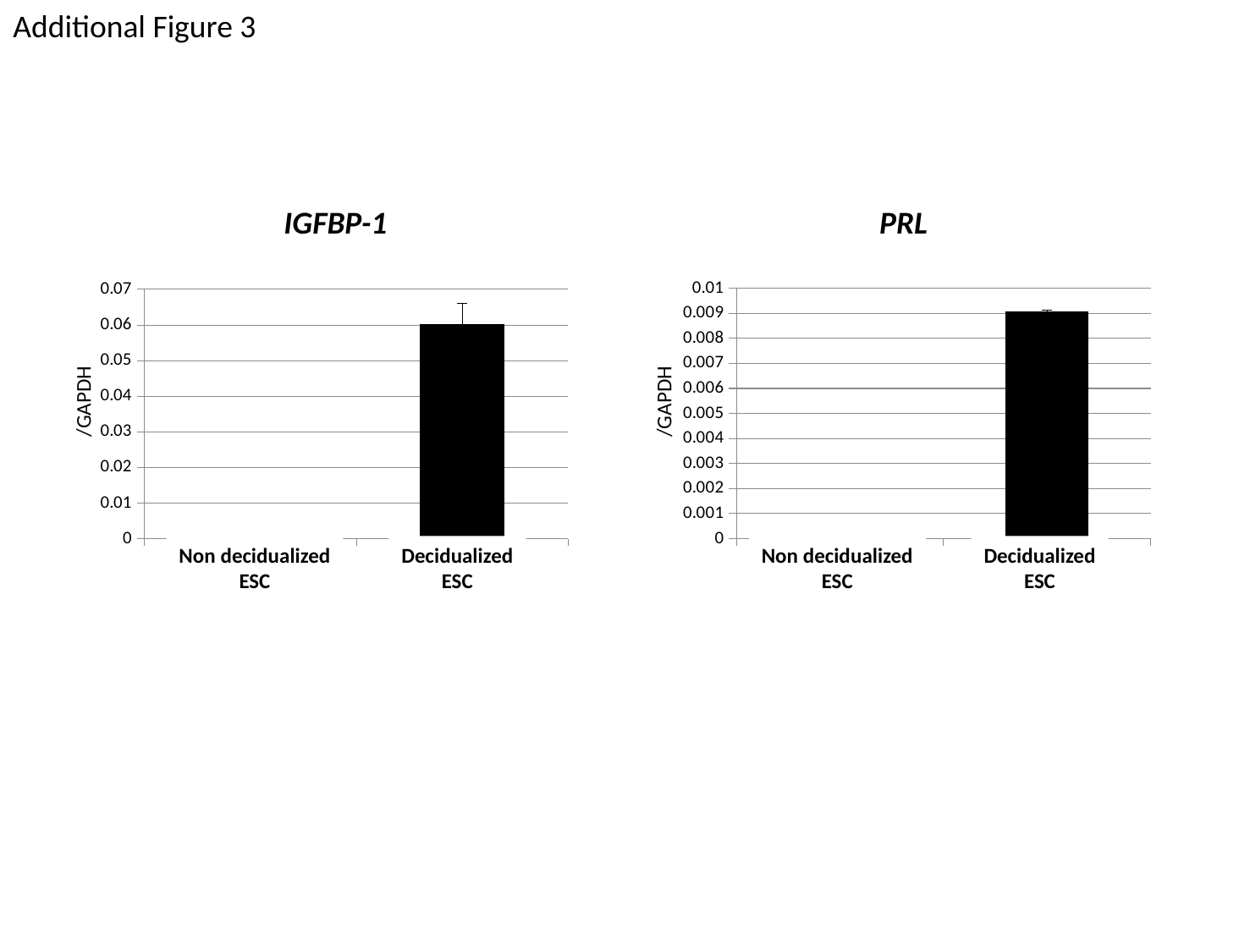

Additional Figure 3
IGFBP-1
PRL
### Chart
| Category | |
|---|---|
| control | 3.329890435356362e-05 |
| E+MPA | 0.00907882002785774 |
### Chart
| Category | |
|---|---|
| control | 1.5020804271023258e-05 |
| E+MPA | 0.06037029749366616 |/GAPDH
/GAPDH
Non decidualized
ESC
Decidualized
ESC
Non decidualized
ESC
Decidualized
ESC
