# Supplementary material for: Correction: Genome-wide DNA methylation analysis revealed stable DNA methylation status during decidualization in human endometrial stromal cells
Source: BMC Genomics. 2024 Apr 5;25:343. doi: 10.1186/s12864-024-10222-4 (PMC10996215; doi:10.1186/s12864-024-10222-4)
Supplement: Supplementary file 4 — Supplementary Material 4 [file 12864_2024_10222_MOESM4_ESM.pptx]

## Slide 1
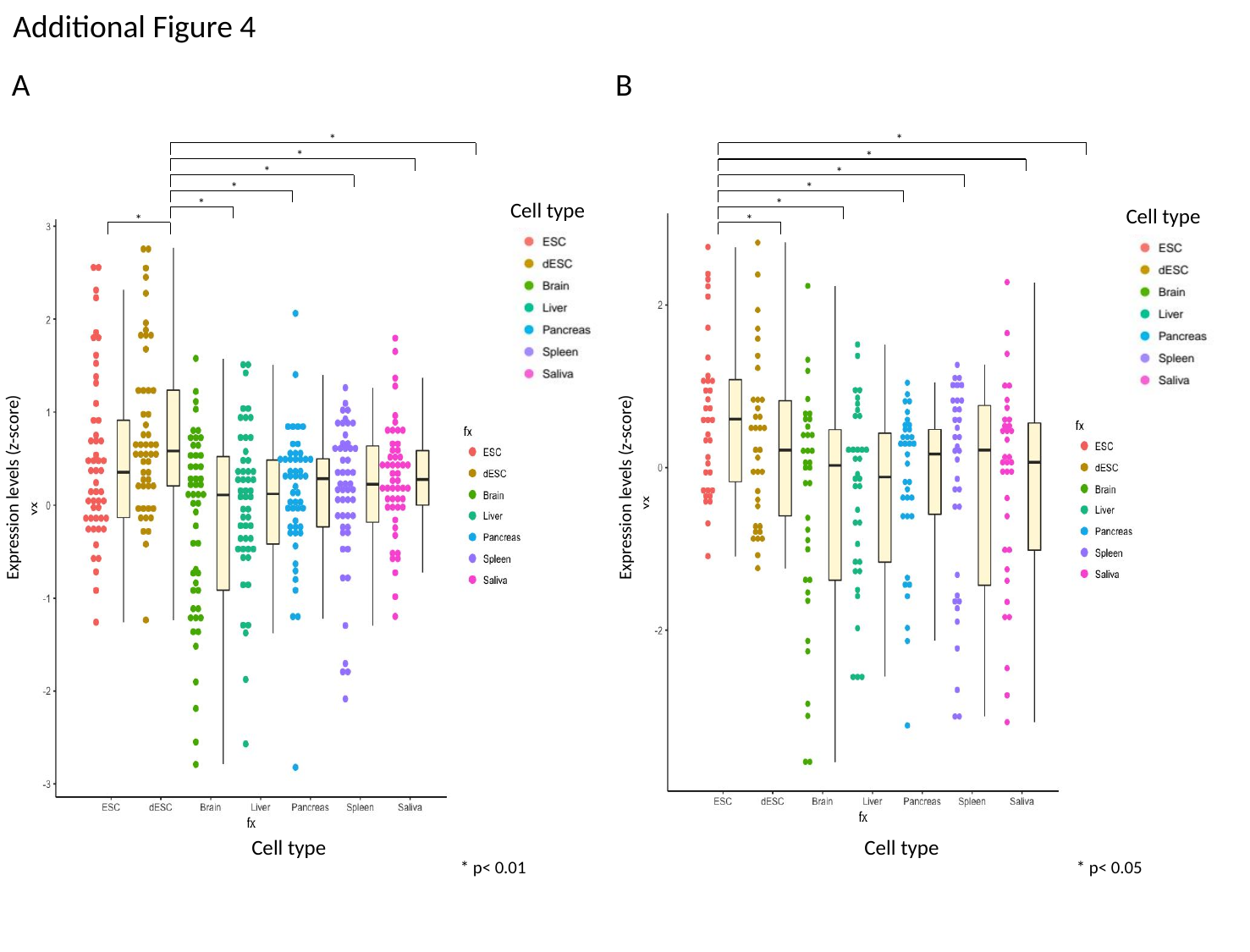

Additional Figure 4
A
B
*
*
*
*
*
*
*
*
*
*
Cell type
Cell type
*
*
Expression levels (z-score)
Expression levels (z-score)
Cell type
Cell type
* p< 0.05
* p< 0.01
